# Loss of a Premature Stop Codon in the Rice Wall-Associated Kinase 91 (*WAK91*) Gene Is a Candidate for Improving Leaf Sheath Blight Disease Resistance

**DOI:** 10.3390/genes14091673

**Published:** 2023-08-24

**Authors:** Noor Al-Bader, Austin Meier, Matthew Geniza, Yamid Sanabria Gongora, James Oard, Pankaj Jaiswal

**Affiliations:** 1 Department of Botany and Plant Pathology, Oregon State University, Corvallis, OR 97331, USA; noormohammedalbader@gmail.com (N.A.-B.); austin.e.meier@gmail.com (A.M.); matthew.geniza@gmail.com (M.G.); 2 Molecular and Cellular Biology Graduate Program, Oregon State University, Corvallis, OR 97331, USA; 3 Louisiana State University Agricultural Center, Baton Rouge, LA 70803, USA; yamid.sanabria@gmail.com (Y.S.G.); joard@agcenter.lsu.edu (J.O.)

**Keywords:** rice, leaf sheath blight resistance, *R. solani*, transcriptome, *WAK91*, wall-associated kinase, plant disease resistance, plant pathology, genomics, double haploid population

## Abstract

Leaf sheath blight disease (SB) of rice caused by the soil-borne fungus *Rhizoctonia solani* results in 10–30% global yield loss annually and can reach 50% under severe outbreaks. Many disease resistance genes and receptor-like kinases (RLKs) are recruited early on by the host plant to respond to pathogens. Wall-associated receptor kinases (WAKs), a subfamily of receptor-like kinases, have been shown to play a role in fungal defense. The rice gene *WAK91* (*OsWAK91*), co-located in the major SB resistance QTL region on chromosome 9, was identified by us as a candidate in defense against rice sheath blight. An SNP mutation T/C in the *WAK91* gene was identified in the susceptible rice variety Cocodrie (CCDR) and the resistant line MCR010277 (MCR). The consequence of the resistant allele C is a stop codon loss, resulting in an open reading frame with extra 62 amino acid carrying a longer protein kinase domain and additional phosphorylation sites. Our genotype and phenotype analysis of the parents CCDR and MCR and the top 20 individuals of the double haploid SB population strongly correlate with the SNP. The susceptible allele T is present in the japonica subspecies and most tropical and temperate japonica lines. Multiple US commercial rice varieties with a japonica background carry the susceptible allele and are known for SB susceptibility. This discovery opens the possibility of introducing resistance alleles into high-yielding commercial varieties to reduce yield losses incurred by the sheath blight disease.

## 1. Introduction

The genetic arms race between pathogens and host plants has been an ongoing tug-of-war for millennia. The “battles” are disruption of pathogen recognition, molecular interactions, signaling, and information transmission on a subcellular level. One such battle occurs between rice, an important crop that feeds 50% of the global population [1], and the soil-borne pathogen *R. solani*, which causes leaf sheath blight disease (SB) [1,2,3,4,5,6]. Leaf sheath blight is a major disease of rice that negatively affects crop yield and quality. Identified by the lesions on the leaf sheath [7,8], SB causes leaves and tillers (secondary shoots) to undergo early senescence, drying out, and tissue death. Ultimately, the significant loss in leaf area due to infection affects the plant’s photosynthetic ability, reducing biomass and yield. No known rice cultivars are fully resistant to leaf sheath blight disease [9,10,11]. Planting high-production rice varieties susceptible to SB in the United States resulted in significant yield losses [6] in a severe breakout season. In 2021, approximately 23% of the rice planting area in the United States was sprayed with fungicide [12], which incurs unsustainable economic costs for farmers [13]. Spraying may also pose severe ecological constraints on animals and microbiomes that coinhabit or frequently visit rice fields [14]. Classified as a soil-borne basidiomycete fungus, *R. solani* is a destructive plant pathogen [15]. In addition to its impact on rice, *R. solani* infects other important crops, such as soybean, barley, sorghum, tomato, and maize [16]. Although *R. solani* rarely produces spores for mobility, its sclerotia can survive in the soil for up to two years [17]. Flooding in paddy fields, as a standard practice or natural occurrence combined with a highly humid environment, causes sclerotia to spread and attach to the plant, causing the disease in susceptible varieties.

**Figure 1:**
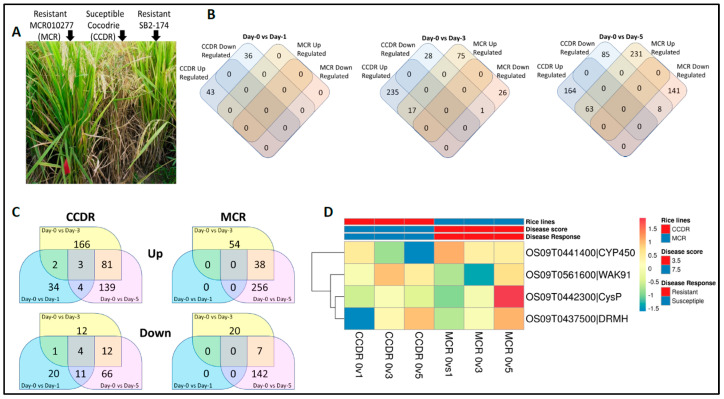
Phenotype and gene expression comparison between the susceptible Cocodrie (CCDR) and the resistant MCR010277 (MCR) rice lines across time points in response to the pathogen Rhizoctonia Solani (strain LR172). (**A**) The rice leaf sheath blight disease response shown by the parent lines MCR and CCDR, and the SB2-174 individual of the SB2 double haploid population. (**B**) The number of differentially expressed genes between the two parent lines when compared to Day 0 vs. Day 1, Day 3 and Day 5 after infection. (**C**) Number of up- and downregulated genes shared between the time points within each rice line. (**D**) The differential expression of four candidate genes overlapping the major leaf sheath resistance QTL on chromosome-9 of rice. Rows are each of the four candidate genes and columns are (L-R) CCDR Day 0 vs. Day 1, Day 3, and Day-5 and MCR Day 0 vs. Day 1, Day 3, Day 5.

Currently, SB disease management relies heavily on fungicide spray [8]. This approach may not be the most sustainable due to potential resistance developing in *R. solani* populations and pollution of agricultural resources [16]. For example, in 2012, a strain of *R. solani,* resistant to the strobilurin class of fungicides used in rice farming, was isolated in Louisiana, USA [18,19]. A more sustainable approach to disease management is to breed genetic resistance to SB in commercial varieties. In this RNA-Seq-based transcriptome study, we compared the two rice lines, a Louisiana variety, SB-susceptible Cocodrie (CCDR, PI 606331) [20], and resistant MCR010277 (MCR, PI 641932) [21] (Figure 1A), examining their response to *R. solani* infection at multiple time points, including Day 0 (untreated control) and Days 1, 3, and 5 after inoculation.

Bioinformatics analysis was used to profile variety- and time-point-specific differential expression of all rice genes, focusing on those found near known SB resistance QTL on chromosome 9 [22,23]. We aligned the transcriptome sequence reads to the reference rice genome to identify variety-specific synonymous and nonsynonymous SNPs and indels (insertions and deletions) and additionally probed their putative consequences on transcript structure, regulation, and protein function in silico. The select set of SNP markers identified in the differentially expressed candidate genes in the major SB-resistance QTL region on chromosome 9 was confirmed by sequencing. The SNP markers were further used for genotyping the resistant and susceptible individuals derived from a biparental (CCDR × MCR) doubled haploid (DH) population of 197 individuals.

We observed clear gene expression and phenotype differences in response to *R. solani* infection in the two rice parental lines. Our findings also reveal SNP markers in the differentially expressed plant defense response genes. Based on the gene expression data, genotyping, and phenotyping, we present strong evidence supporting a rice *Wall Associated Kinase 91* (*WAK91*, *OsWAK91*) gene as a potential breeding target for developing SB-resistant breeding lines.

## 2. Materials and Methods

### 2.1. Plant Material and Inoculation

We planted the susceptible variety Cocodrie (CCDR, PI 606331) [20] and the sheath blight-resistant rice line MCR010277 (MCR, PI 641932) [21] in pots placed in a mist chamber in a greenhouse located at the Louisiana State University (LSU) campus in Baton Rouge, LA, USA. Temperatures inside the mist chamber ranged from a minimum of 27 °C at night to a maximum of 37 °C during the day. Natural daylight was used, with a day length of approximately 11 h 30 min. Humidity was maintained at 80–90% using a Vicks cool mist humidifier of 1.2 gallons capacity programmed to function for two hours every six hours. We constructed the chamber frame with ¾ inch PVC pipe and covered it with extra light plastic (0.31 mm) (Painter’s Plastic sheet). The chamber dimensions were 1.32 m wide by 2.70 m in length and 1.42 m in height, for a total capacity of 48 pots per chamber, each containing three plants.

Plants were inoculated 50 days after germination with a Potato Dextrose Agar PDA medium disc (0.8 cm diameter) containing *R. solani* (strain LR172) mycelia. Discs were placed at the base of the stem and between the leaf blade and leaf sheath on the main culm of each plant. Leaf samples approximately 2 cm in length were collected from the control untreated (day 0) and inoculation sites 1, 3, and 5 days after treatment and placed immediately in liquid nitrogen until transferred to a −80 °C freezer for storage.

### 2.2. RNA Extraction and Sequencing

Frozen leaf samples from the CCDR and MCR lines collected at LSU were shipped to Oregon State University on dry ice and stored at −80 °C—until further processing. Poly(A)-enriched mRNA libraries were prepared from total RNA extracted from the leaf samples using RNA Plant Reagent^®^ (Invitrogen, Thermo Fisher Scientific, Carlsbad, CA, USA), RNeasy kits (Qiagen LLC, Germantown, MD, USA), and RNase-free DNase (Life Technologies Inc., Carlsbad, CA, USA). The concentration and quality of the poly(A)-enriched mRNA were determined using an ND-100 spectrophotometer (Thermo Fisher Scientific Inc., Carlsbad, CA, USA) and Bioanalyzer 2100 (Agilent Technologies Inc., Santa Clara, CA, USA), respectively. TruSeq RNA Sample Preparation kits (Illumina Inc., San Diego, CA, USA) were used to construct sequencing libraries. An Illumina HiSeq 3000 (Illumina Inc., USA) at the Center for Genome Research and Biocomputing, Oregon State University (CGRB, OSU), was used to sequence the 150 bp Paired End cDNA libraries.

### 2.3. Sequence Quality Control and Read Alignments

The program Sickle v1.33 was used to filter all reads based on read quality. Reads under the phred score of 30 and with read length under 150 bp were rejected. FASTQ files containing quality-trimmed and filtered reads were generated, yielding high-quality MCR and CCDR reads. Any reads showing alignment to the pathogen *R. solani* AG1-IB isolate (NCBI accession #GCA_000832345.1) were also filtered out. Quality-filtered reads were aligned to the reference *Oryza sativa* japonica cv Nipponbare (IRGSP v1.0) genome and annotation [24]. Reads from each biological replicate were aligned with the alignment software program STAR v.2.4.1a [25]. The resulting sequence alignment files were converted to binary alignment files (BAM), sorted coordinately, and indexed using SAMTools v.13 [26].

### 2.4. Differential Gene Expression (DGE)

The program DESeq2 (v 1.22.2) was used to investigate gene expression levels of the two rice lines over the time course [27]. A Python package, HTSeq v.0.6.1p1 [28], indexed the alignment BAM files and generated raw counts with a minimum quality-score cutoff of 10. The DESeq2 R-package was used to identify differentially expressed genes. First, the raw count data was transformed using the variance stabilizing transformation (vst) method of DESeq2 to filter outliers. The DESeq2 functions, namely estimate size factors and dispersions, were used to normalize the aligned read counts. The DESeq2 function followed this to fit a negative binomial general linear model and Wald test statistics to detect the significance scores (*p*-value). The final set of statistically significant differentially expressed transcripts were called using Benjamini–Hochberg adjusted false discovery rates (FDR) of 10% and cutoff *p*-value ≤ 0.05. Furthermore, the non-significant events identified with the DESeq2 software were filtered out.

### 2.5. SNP Discovery and Variant Prediction

Indexed BAM files were analyzed to identify genetic variations, including single nucleotide changes (SNPs; transitions and transversions) and insertions and deletions (indels). Alignments from each variety were pooled using the mpileup function of SAMtools [26]. VarScan.v2.3.9 (release 80) [29] was used to identify SNPs and indels with four different minimum read coverages of categories of 2, 6, 8, and 20 and a default minimum variant frequency of 0.8 with a *p*-value of 0.005. A consensus set of SNPs was identified in all four minimum read coverages. The Variant Effect Predictor (VEP) workflow provided by the Gramene database [30] was used to infer the putative consequences to the structure, splicing, and function of the gene product (transcript and peptide) based on synonymous and nonsynonymous (ns) changes.

To develop genetic markers from significant SNPs overlapping the major SB-resistance QTL on chromosome 9, we queried the SNP data to find associations with the differentially expressed candidate genes. Additional data on the 3000 rice genomes provided by the Rice SNP Seek project [31,32,33] and genome sequences of the wild species of Oryza available at the Gramene database [34,35] were mined for the same SNP sites to profile the allelic nature and distribution in the diverse rice lines. Finally, we mined the peer-reviewed literature to extract and confirm information on the known leaf sheath blight resistance phenotype for these rice lines.

### 2.6. Population Study

The RiceCAP SB2 mapping population was developed as a genetic resource to identify lines containing molecular markers associated with SB resistance [36]. The SB2 population consists of 197 doubled-haploid (DH) lines derived from a cross between the susceptible parent Cocodrie (CCDR, PI 606331) [20] and the resistant parent MCR010277 (MCR, PI 641932) [21]. The population and the two parents were evaluated for response to leaf sheath blight disease on a scale of 0–9, where 0 = no disease and 9 = dead plant [7,8,37]. Plants were grown and phenotyped across three years at Crowley, LA, USA, and Stuttgart, AR, USA [37]. The top ten SB-resistant individuals, SB2-03 (GSOR200003), SB2-109 (GSOR200109), SB2-134 (GSOR200134), SB2-158 (GSOR200158), SB2-161 (GSOR200161), SB2-174 (GSOR200174), SB2-206 (GSOR200206), SB2-225 (GSOR200225), SB2-259 (GSOR200206), and SB2-272(GSOR200272 and the ten most susceptible individuals, SB2-13 (GSOR200013), SB2-48 (GSOR200048), SB2-88 (GSOR200088), SB2-99 (GSOR200099), SB2-125 (GSOR200125), SB2-144 (GSOR200144), SB-203 (GSOR200203), SB-255 (GSOR200255), SB-276 (GSOR200276), and SB2-314 (GSOR200314) from the SB2 DH population, and the two parents were screened with 130 candidate SNP markers (Supplementary Tables S3 and S5) identified by this work and Silva et al., 2012 [21]. One-way ANOVA analysis was performed to calculate the F values, Bonferroni false discovery rates (FDR), *p*-values, and adjusted R^2^ values.

### 2.7. Sampling DNA from Rice Varieties

Plants from 13 rice lines: CL153, Cypress, Blue Bonnet, CL111, 93-11, IR29, IR64, Jasmine, TeQing, Pokkali, Nonabokra, and AUS lines Kasalath and Nagina 22 (N22) were grown in the Oregon State University West greenhouses using the same growth conditions as mentioned earlier. The leaves from 10-day old plants were collected for DNA extraction and SNP marker genotyping via DNA amplification and sequencing. DNA was extracted using a DNeasy Plant Mini Kit (Qiagen Inc., San Diego, CA, USA). PCR primers were bought from Invitrogen Inc., USA, to amplify the region of interest (Supplementary Table S6). We used Ready PCR Mix, 2× (VWR), for PCR reactions. The C100 Thermocycler (BioRad) was set for 3 min at 95C temperature for initial denaturation, followed by 40 cycles of 30 s at 95 °C for denaturation, 30 s at 55 °C for annealing, 30 s at 72 °C for extension, and 5 min at 72 °C for final extension. Amplified DNA fragments were separated on a 1% agarose gel in 1× TAE buffer. These DNA fragments were extracted from the gel using QIAGEN Gel Extraction and QIAGEN PCR product cleanup kits. Isolated PCR products were Sanger sequenced at the CGRB, OSU. The amplified sequences from the PCR products for each rice line, including the parents MCR and CCDR, were aligned to the chromosomal region of the reference Nipponbare genome (IRGSP v1.0) using ClustalW to confirm SNPs and identify the alleles.

### 2.8. Data Mining and Functional Annotation

The reference genome sequences, gene function, and gene family annotations were accessed from the Gramene database (www.gramene.org accessed, 2018) [35,38]. The 3000 rice genome project data was mined at Rice SNP-Seek Database (http://snp-seek.irri.org/ accessed on 22 May 2018) [31]. Gramene database was queried to pull out all rice genes (IRGSP v1.0) annotated to carry the WAK domain and that were part of the WAK gene family. The protein sequences of the canonical (longest) isoform were downloaded in the fasta format to run the ClustalW alignment with default parameters. The gene family tree and the differential gene expression data for the respective rice WAK gene family members were uploaded on the iTOL web portal [39] to generate the graphics.

## 3. Results

### 3.1. Transcriptome Analyses

We prepared twenty-four cDNA libraries from three replicates and four time points for each rice line. The sampled time points were Day 0 (untreated control) and Days 1, 3, and 5 after *R. solani* inoculation. Strand-specific 150 bp paired-end sequencing of the cDNA libraries yielded 308,482,684 (CCDR) and 357,018,002 (MCR) reads. After filtering the low-quality raw reads, we obtained 299,687,236 and 346,498,184 (~98.5% of total) sequence reads from the CCDR and MCR samples, respectively, with an average of 27.7 million reads per sample. The sequence reads were aligned to the *O. sativa japonica* cv Nipponbare (IRGSP v1.0) reference genome. A total of 16,480 CCDR and 17,793 MCR protein-coding genes showed normalized expression at least at one time point. The expression of known genes from each line on Days 1, 3, and 5 were compared against the untreated Day 0 sample for the differential gene expression analysis. For CCDR, we identified 79, 281, and 320 differentially expressed genes on Day 1, Day 3, and Day 5, respectively. In the MCR line, we identified 119 and 443 genes on Day 3 and Day 5, respectively. We did not observe any statistically significant differences in expressed genes on Day 1 in the MCR line (Figure 1B). On Day 3, 17 common genes were upregulated in both the CCDR and MCR lines and 1 was downregulated. Similarly, on Day 5, 63 common genes in the CCDR and MCR lines were upregulated, while 8 genes were downregulated (Figure 1B). In the susceptible CCDR line, there were more differentially expressed genes on Days 1–5, unlike the MCR line, which had more significant numbers of differentially expressed genes on Days 3 and 5 (Figure 1C).

In the CCDR line, genes with transcription factor function and those that play a role in the biotic stress response were upregulated at Day 1. Similarly, upregulated genes showed enrichment of hydrolase enzyme function at Day 3 in the CCDR line. One gene (OS02G0129800), which is grass (Poaceae) family-specific, was downregulated in both the CCDR and MCR lines on Day 3. This gene on chromosome 2 was upregulated in response to *Burkholderia glumae* [40], the causal agent of rice bacterial panicle blight disease. In contrast, it was downregulated in response to *Xanthomonas oryzae*, the causal agent of rice bacterial leaf streak disease [41]. Day 5 showed the enrichment of genes associated with the catabolism of peptidoglycan, an important cell wall component, those associated with biotic stimulus and defense responses, kinases, and enzymes with chitinase activity required for degrading the fungal pathogen cell walls.

We identified four differentially expressed candidate genes in the vicinity of the major SB resistance QTL on chromosome 9: a monocot lineage-specific dormancy auxin-associated family protein (DRMH, OS09G0437500), an elicitor-inducible cytochrome P450 (CYP450, OS09G0441400), a cysteine peptidase (CysP, OS09G0442300), and a *Wall-Associated receptor kinase 91* (*WAK91*, *OsWAK91*, OS09G0561600), with contrasting expression profiles (Figure 1D). The *OsWAK91* transcript showed downregulation up to Day 3, followed by upregulation at Day 5 in the MCR line compared to being constitutively expressed in the CCDR line. OS09G0441400, the elicitor-responsive CYP450 gene, showed an expression profile opposite that of *WAK91*. OS09G0437500, the dormancy auxin-associated gene, exhibited a similar expression profile between the two lines. In contrast, OS09G0442300, a cysteine peptidase, was highly expressed in the MCR on day 5.

### 3.2. SNP Marker Discovery and Variant Cause Prediction

**Figure 2:**
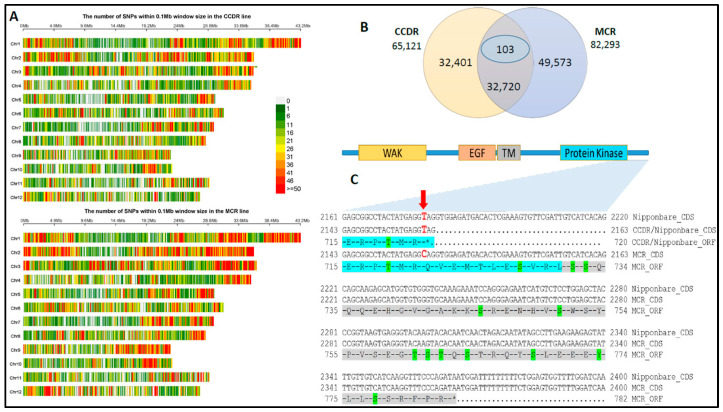
Distribution of SNP sites (including insertions and deletions) identified in the susceptible CCDR and the resistant MCR rice lines and their consequence on the rice *WAK91* gene. (**A**) Density of SNP sites in the CCDR and the MCR lines mapped across the reference japonica rice genome (IRGSP v1.0). (**B**) Number of unique and shared SNPs identified in the two rice lines. (**C**) The rice *WAK91* ORF showing the regions of the functional domains (**top**) and zoom-in view of the CDS and the ORF from the reference Nipponbare and parents CCDR and MCR rice lines (**bottom**). * Stop codon; open reading frame in the kinase domain region (cyan colored); potential phosphorylation sites (green colored).

We used the transcriptome sequence read alignments to identify 65,121 and 82,293 genetic variants in the susceptible CCDR and resistant MCR lines, respectively. These variants include single nucleotide changes (SNPs) in the form of transitions, transversions, insertions, and deletions (indels). The SNP sites were distributed across the rice genome with varying degrees of density when compared between the two lines (Figure 2A). We found that 32,401 and 49,573 SNPs were unique to CCDR and MCR, respectively, and they shared 32,720 SNPs present at the same loci. Of the shared loci, 103 SNP sites contained alleles different from the reference (Nipponbare) and between the two lines (Figure 2B). On average, we observed that the MCR line carried ~2500 transitions (Ts), ~750 transversions (Tv), and ~1000 more indel SNP events than the CCDR line (Table 1). TvSNPs have more significant regulatory effects than TsSNPs [42], and rice is known to carry more of the transition types of SNPs [43]. The TsSNPs were more significant in number in both lines, but the transition to transversion ratio (Ts/Tv) in the MCR line was 2.8 compared to 2.68 in CCDR. Within the TsSNPs, there was almost the same number of A⟷G and T⟷C transitions in CCDR compared to MCR, whereas there were more G→A and C→T transitions than A→G and T→C, respectively. The transition type G→C was high in CCDR compared to MCR, where the opposite type C→G was more abundant. MCR had significantly fewer C→A TsSNPs (Table 1).

The identified genetic variants mapped to 22,448 and 24,343 rice reference protein-coding genes in the CCDR and MCR lines, respectively, constituting 55% and 66% of the genes in the published reference rice genome [24]. Because we used the cDNA sequence reads for SNP calling and genetic marker development, we used the Variant Effect Predictor (VEP) tool [30] to predict the causal effect of each SNP on the 5′ and 3′ UTRs, exons, intron, and intron splicing boundaries of the transcribed gene. If the SNP was in an exon, we also computed the synonymous (sSNP) and nonsynonymous (nsSNP) variations to predict the putative consequences on transcript structure, regulation, splicing, and peptide structure and function (Supplementary Table S1).

**Figure 3:**
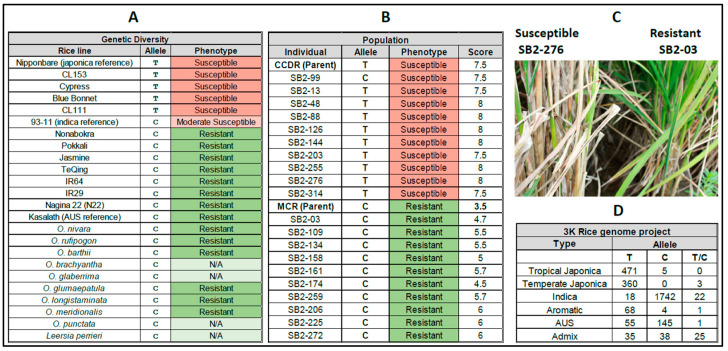
Genotype, sequence and phenotype information on various Oryza species, rice lines and the SB2 double haploid population. (**A**) Rice *WAK91* SNP allele T/C in 13 rice lines was confirmed by sequencing the SNP region. Synteny-based sequence data were mined at the publicly available Gramene database for the reference lines indica 93-11, japonica Nipponbare, the wild species of the Oryza genus and its outgroup Leersia Perrieri. The leaf sheath blight disease response phenotypes were mined from previously published literature. (**B**) Genotyping and phenotyping of the top ten resistant and ten susceptible individuals of the SB2 double haploid population. The population and the two parents were evaluated for sheath blight response phenotype on a scale of 0–9, where 0 = no disease and 9 = dead plant. The *WAK91* SNP alleles T/C were scored for presence/absence. (**C**) The leaf sheath blight response phenotype of the individuals SB2-276 (susceptible) and SB2-03 (resistant) from the DH population. (**D**) Occurrence of the *WAK91* SNP allele T/C as reported in the genomes of 3000 rice lines and their grouping.

We developed genetic markers associated with the disease resistance phenotype in the population study based on these VEP and differential gene expression results. The set of 130 SNP genetic markers [21], including those identified herein, was short-listed by selecting chromosomal locations overlapping the major and minor SB QTLs. Previously identified sheath blight QTLs are present on chromosomes 1, 2, 3, 4, 5, 6, 7, 8, 9, 11, and 12 [22,37,44,45,46,47,48,49,50,51,52]. In the major SB QTL region on chromosome 9, we found that the differentially expressed *WAK91* (OS09G0561600) gene carried a nonsynonymous SNP (T→C) aligned at position 22,318,449 bp of the Nipponbare rice reference genome (IRGSP v1.0; Figure 2C). The SB-susceptible line CCDR carried the same allele T as the reference, whereas the resistant MCR line carried the C allele. The T→C transition results in the loss of a stop codon in the MCR *WAK91*, resulting in a predicted *WAK91* peptide with an additional 62 amino acids. Sequencing the amplified region of the *WAK91* SNP marker confirmed the presence of the susceptible T allele in the parent CCDR, as well as other US elite lines with a japonica background, namely CL53, Cypress, Blue Bonnet, and CL111, whereas in the indica lines, IR29, IR64, Jasmine, TeQing, Pokkali, Nonabokra, 93-11, and the AUS lines Kasalath and Nagina22 (N22) were confirmed to carry the resistant allele C (Figure 3A).

### 3.3. Double Haploid Population Study

Evaluation of the ten most resistant individuals of the SB2 double haploid (DH) population produced disease ratings between 4.7 and 6.0, while the resistant parent MCR showed a 3.5 rating in the same study. In contrast, the ten most susceptible individuals of the population scored between 7.5 and 8.0, while the susceptible parent CCDR had a 7.5 rating (Figure 3B,C, Supplementary Table S2). Selective genotyping of the MCR and CCDR parent lines and 20 selected DH lines was performed with 130 SNP markers in the SB QTL regions on chromosomes 1, 2, 3, 4, 5, 6, 8, 9, 11, and 12. These marker sites included the nonsynonymous *WAK91* SNP (T/C) identified in this study. The ANOVA analysis identified 11 best-ranked SNP markers. These markers mapped to the bottom of chromosome 9 overlapping the SB resistance QTL region identified previously in 12 separate studies using six indica lines (reviewed by Zuo et al. [53,54]). The genotyping data showed that the *WAK91* SNP allele C contributed by the MCR parent line always cosegregated with the SB resistance phenotype, except in the SB2-99 line. In contrast, allele T from the CCDR parent cosegregated with the susceptible phenotype (Figure 3B).

## 4. Discussion

In our quest to find candidate genes in rice that can provide a good measure of resistance to the pathogen *R. solani* and their application for breeding improved disease-resistant rice, our integrated transcriptomics study identified genetic variation and its causes of gene function along with association to sheath blight disease resistance. We identified the rice candidate gene *Wall-Associated Kinase 91* (*WAK91*) carrying a T/C mutation at the stop codon. The *WAK91* SNP site at position 22,318,449 bp on chromosome 9 of the reference rice genome is part of the stop codon (TAG), and allele T is common to both the CCDR and the reference japonica line Nipponbare. Due to the presence of allele C in the resistant line MCR, the stop codon TAG is lost and replaced by the codon CAG in the *WAK91* open reading frame, which codes for glutamine, a polar uncharged side chain amino acid. The stop codon loss in the MCR line also results in the *WAK91* peptide that is 62 amino acids longer than in the CCDR and Nipponbare (Figure 2C). These results lead to two hypotheses: (1) the evolutionarily conserved *WAK91* SNP allele C is associated with the SB resistance phenotype, and (2) the longer *WAK91* ORF resulting from the loss of the stop codon results in a peptide with a gain in novel molecular function that may play a role in leaf sheath blight resistance. Overexpression of the rice gene *Broad Spectrum Resistance 2* (*BSR2*, Os08g0547300) was known to confer resistance to SB [4], and we found it highly expressed in the resistant MCR line on Day 1, followed by subsequent reduction in expression on Days 3 and 5. The CCDR line showed 20% less expression on Day 1 and downregulation on Days 3 and 5. BSR2 is an uncharacterized cytochrome P450 protein-coding gene belonging to the CYP78A family

To test the evolutionary conservation of the *WAK91* SNP allele C with SB resistance, we sequenced a PCR-amplified fragment overlapping the *WAK91* SNP loci from 13 additional *O. sativa* lines: five from subspecies japonica and eight from subspecies indica. All the US elite rice lines carry the japonica background and are known to be SB-susceptible [22]. We predicted and confirmed that the US japonica-genetic-background-containing lines CCDR, CL53, Cypress, Blue Bonnet, and CL111 carry the *WAK91* SNP allele T associated with known susceptibility phenotype [10,55,56,57,58,59,60,61,62]. In contrast, the indica lines IR29, IR64, Jasmin, TeQing, Pokkali, and Nonabokra, and AUS lines Kasalath and Nagina 22, were confirmed to carry the resistant C allele (Figure 3A). These indica and AUS lines exhibit some known SB resistance phenotypes [53,54,59,63,64]. One exception is the rice line 93-11 with the indica reference genome [65], which is known to be moderately susceptible [53,66]. These results are consistent with the genotype and phenotype observations of the 20 individuals of the SB2 DH population, except for sibling SB2-99 (Figure 3B,C, Supplementary Table S2).

To gain insights into the evolutionary significance and trace the origin of the *WAK91* SNP in the *Oryza* genus, we used synteny data provided by the Gramene database [35,38]. We aligned the SNP region between the sequenced genomes of the wild species *O. longistaminata*, *O. glaberrima*, *O. punctata*, *O. meridionalis*, *O. barthii*, *O. glumaepatula*, *O. rufipogon*, *O. brachyantha*, and the outgroup *Leersia perrieri*. These species carry the indica-type allele C, and most are known to bear the SB resistance phenotype (Figure 3A) [57,67,68,69]. It suggests that the indicia-type resistant C allele is of ancestral origin. Conversely, the japonica-type susceptible T allele is a recent introduction in the *Oryza* clade, and its origin appears in the *O. sativa subspecies japonica*. We confirmed its origin by mining publicly available genetic variation data from the 3000 Rice Genome Project [33] available from the RiceSNP-seek database [31]. Most temperate and tropical japonica and aromatic rice lines carry the japonica T allele, whereas most indica and AUS lines carry the C allele. Only a handful of the lines carried the heterozygous T/C allele (Figure 3D). We did not have access to the SB resistance phenotype data for these lines; however, based on our observations, we predict that the indica and AUS lines carrying the C allele may bear some degree of SB resistance.

**Figure 4:**
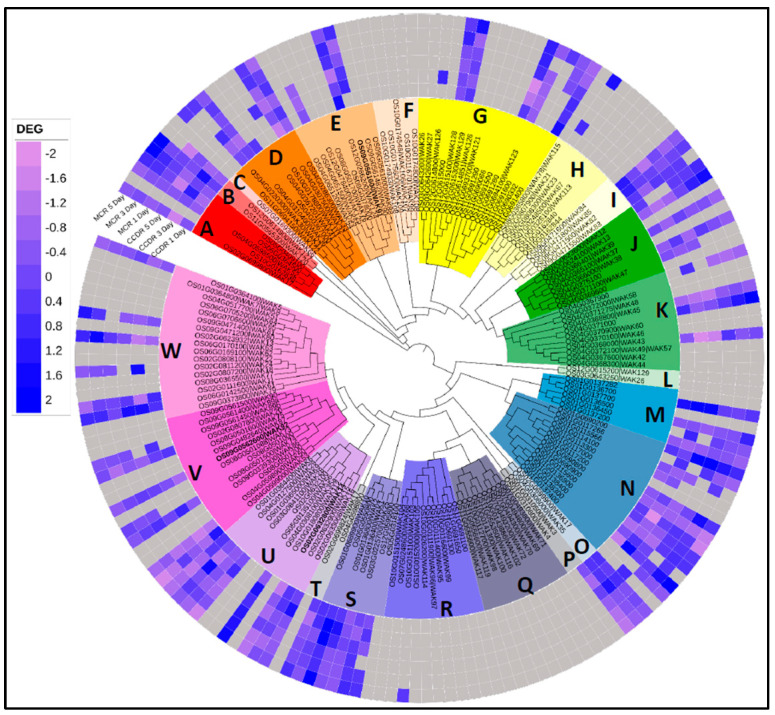
Phylogenetic tree of the rice wall. Associated kinase gene family members distributed in 23 subfamilies. Differential gene expression data from the MCR and CCDR rice lines were plotted in the track order (outside to inside) MCR Day 0 vs. Day 5, Day 3, and Day 1 and CCDR Day 0 vs. Day 5, Day 3 and Day 1.

The second hypothesis suggests that the *WAK91* gene from the SB-resistant MCR parent encodes a peptide predicted to be 62 amino acids longer due to stop loss in the ORF, and this peptide may have gained a function that plays a role in providing the SB resistance phenotype. The *WAK91* gene is a member of the wall-associated receptor kinase (WAK) gene family (Figure 4) and encodes a plasma membrane protein. The *WAK91* protein contains a wall-associated receptor kinase galacturonan-binding domain (WAK domain), followed by a calcium-binding epidermal growth factor (EGF)-like domain in the N-terminal half of the protein that is present on the extracellular side. In the middle is a single transmembrane domain spanning the plasma membrane, followed by the C-terminal cytosolic half containing a protein kinase domain and additional phosphorylation sites for serine, threonine, and tyrosine residues (Figure 2C). The WAK domain is known for linking to the pectin fraction of the plant cell wall [70,71,72].

The WAK gene family members are known to express and function in response to biotic and abiotic stress conditions. Expression of *Arabidopsis thaliana AtWAK1* is known to be induced by pathogen response simulated by exogenous salicylate or by its analog 2,2-dichloroisonicotinic acid and requires a positive regulator NPR1/NIM1. The expression of complete AtWAK1 or its cytoplasmic kinase domain alone can resist lethal salicylic acid levels [73,74]. Maize wall-associated receptor-like kinase *Htn1*, a plasma membrane protein, is known for conferring resistance to northern corn leaf blight disease caused by the fungal pathogen *Setosphaeria turcica* (anamorph *Exserohilum turcicum*, previously known as *Helminthosporium turcicum*) by restricting pathogen entry into host cells [75,76]. Similarly, the wheat WAK gene *Stb6* confers fungal pathogen resistance without a hypersensitive response [77]. Barley, *HvWAK1* is known to play a role in root development; however, compared to other cereals and Arabidopsis, sequence divergence in the extracellular domain further verifies the multifunctionality of WAK genes [78]. WAKs also play roles in plant cell expansion during seedling development and MAP kinase signaling; they bind to pectin polymers in the plant cell wall and have a higher affinity to bind smaller pectin fibers in response to pathogen attack [70]. 

In the reference rice genome, the WAK gene family members are distributed in 23 subfamilies (Figure 4) and are reported to play roles in development [79], abiotic responses [80,81], and fungal disease responses [82]. Our study did not find expression of genes from five WAK subfamilies, C, F, L, and Q (Figure 4). The gene *OsWAK1* (OS01G0136400) is known to provide resistance against the rice blast disease pathogen *Magnaporthe grisea*, and its salicylic acid and methyl jasmonate induce its expression. *OsWAK1* phosphorylates itself and OsRFP1, a putative transcription regulator that binds to the *OsWAK1* kinase domain [83]. We observed downregulation of *OsWAK1* at all time points except in the CCDR line on Day 5. The candidate gene *WAK91* gene is also known as *DEFECT IN EARLY EMBRYO SAC1* (*OsDEES1*) [84]. *WAK91* and the *OsWAK1* play roles in rice sexual reproduction by regulating the development of the female gametophyte (the embryo sac) and playing a role in anther dehiscence, respectively [79,84,85]. Overexpression of *OsWAK25* (OS03G0225700) is known to increase susceptibility to *R. solani* and *Cochliobolus miyabeanus* and resistance to X. oryzae pv. *oryzae* (*Xoo*) and *Magnaporthe oryzae* [86], whereas its loss of function compromises Xa21-mediated resistance [87,88]. We observed upregulation of *OsWAK25* at all time points except for downregulation on Day 3 in the CCDR line. Other gene family members, such as *OsWAK11*, regulate copper detoxification [80,81], and *OsWAK112* negatively regulates the salinity response by suppressing ethylene production [67].

**Figure 5:**
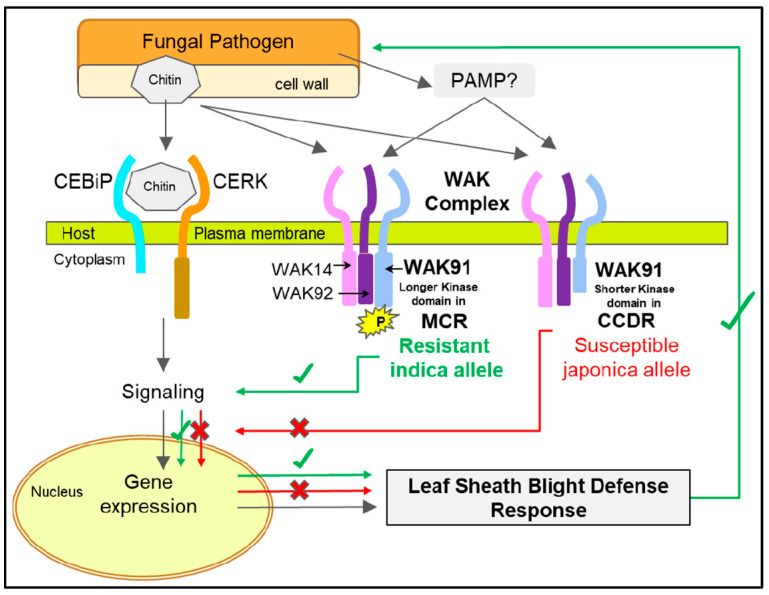
A hypothetical model of the host. Pathogen interaction showing the response to fungal chitin binding by the chitin binding receptor complex and po-tential interactions and functions by the heterotrimeric wall-associated kinase (WAK) receptor protein complex. The WAK complex is functional in both the MCR and the CCDR rice lines; however, the MCR *WAK91* C-terminus carrying the longer protein kinase domain and the additional serine, threonine and tyrosine phosphorylation sites is expected to play a role in successfully initiating the downstream signaling response to provide leaf sheath blight resistance.

Overexpression of *WAK91* is known to increase rice blast resistance and reduce fungal hyphal growth. In contrast, overexpression of *OsWAK112* (OS10G0180800) suppressed resistance [84,85]. The genes *OsWAK14* (OS02G0632800), *WAK91*, and *OsWAK92* (OS09G0562600) are part of the heterotrimeric WAK protein complex located on the plasma membrane in rice cells [89,90] (Figure 5). A mutant screening by Delteil et al. [82] reported that the loss of function of *OsWAK14*, *OsWAK91*, and *OsWAK92* resulted in reduced basal resistance, although it did not affect growth and fertility [90]. Conversely, we found that the CCDR and Nipponbare rice reference lines carry the susceptible T allele with a shorter *OsWAK91* ORF and are known for SB susceptibility. We hypothesize an increased pathogen susceptibility if the *WAK91* function is curtailed or when the longer C-terminal is absent (such as in the CCDR and Nipponbare). Therefore, we can expect that the Nipponbare *WAK91* knockout line would be highly susceptible. Although not tested for response to *R. solani*, Deltiel et al. [82] confirmed that wild-type Nipponbare (a susceptible line) and the *WAK91* Tos-17 mutant plants always showed increased disease lesions in response to the pathogen *M. grisea*, the causal agent of rice blight disease. The *WAK91* mutant plant showed a 2.5-fold increase in lesions.

The ability of host plants to detect and fight pathogenic microbes is a complex process. The pattern-recognition receptors (PRRs) localized on the surface of plant cells are well-known gene family members of receptor-like kinase (RLK) proteins that assist in recognition of pathogen-associated molecular patterns (PAMPs), conserved motifs from pathogens, and damage-associated molecular patterns (DAMPs) derived from damage caused by microbial development [90,91,92]. Additionally, in the case of the *M. grisea* blast disease response in rice, the chitin elicitor binding protein OsCEBiP and a chitin elicitor receptor kinase, OsCERK, are known to form a heterodimer complex. Upon chitin binding, the CEBiP-CERK receptor complex may induce transphosphorylation and activation of downstream signaling [93] (Figure 5). In our transcriptome data, most of the OsCEBiP and OsCERK gene family members showed positive upregulation in the resistant MCR line (Supplementary Figure S1).

Similarly, OsWAKs show early transcriptional regulation induced in response to chitin, a process controlled by its receptor CEBiP [92,93] A query of publicly available SNP datasets [31,33,35,94,95] suggests that *WAK91* is known to carry ~400 unique genetic variations present in the 5′ and 3′ UTRs, introns, exons, and splice junctions. It includes the stop loss SNP identified by us, an indel at 22,315,774–22,315,791 bp leading to start loss and 5′UTR variant, and stops gained at positions 22,316,748 bp, 22,317,724 bp, and 22,318,006 bp on chromosome 9, respectively (Supplementary Table S4). These genetic differences did not appear in our transcriptome-based SNP dataset and are potential genetic markers for further study. Therefore, based upon the supporting evidence from the genotyping, phenotyping, evolutionary ancestry of SB tolerance, and the favorably segregating SNP in the SB2 DH population, we propose a working model (Figure 5) in which the candidate gene *WAK91* may provide broad tolerance to leaf sheath blight disease similar to when infected with pathogens *R. solani* and *M. grisea*. The longer ORF of *OsWAK91* in the indica lines and the MCR genotype provides a potentially complete kinase domain and additional active sites for phosphorylation (Figure 2C). *OsWAK91* forms a heterotrimeric protein complex with the *OsWAK14* and *OsWAK92* proteins and is an essential member of the WAK protein complex [90]. The *OsWAK91* in the japonica background with a truncated C-terminus kinase domain is not completely dysfunctional. It still functions in normal embryo sac development since the japonica line Nipponbare and others mentioned in this work are fertile lines.

## 5. Conclusions

The genotyping and phenotyping study of the ten most resistant and ten most susceptible individuals from the double haploid rice population derived from the CCDR and MCR parents inferred a strong association between the rice *WAK91* SNP marker and the leaf sheath blight resistance. We also mined the publicly available sequenced genomes of reference and ancestral *Oryza* species and the 3000 Rice Genome Project to confirm the evolutionary source of the mutant *WAK91* SNP. The resistant allele appears ancestral, whereas the susceptible allele is a more recent acquisition in the *O. sativa japonica* clade. All US elite rice varieties with japonica genetic backgrounds are known to carry the susceptibility trait. We tested a few of them and confirmed the presence of the susceptible SNP allele, similar to the CCDR line. Our results and inferences, supported by sequencing, genotyping, and phenotyping experiments, are well complemented by mutant/knockout screening by earlier studies [82,90]. *WAK91* knockout mutations make rice susceptible to disease. Therefore, the *WAK91* gene with the resistance allele identified by us is a candidate for integration into existing and newly cultivated rice varieties for leaf sheath blight resistance, which may help improve global rice production by rescuing crops affected by the pathogen *R. solani* [23,96].

## Figures and Tables

**Table 1:** Transition and transversion type SNPs and indels identified in the susceptible CCDR and the resistant MCR rice lines by aligning the RNA-Seq sequence reads against the reference *O. sativa japonica* cv Nipponbare reference genome (IRGSP v1.0).

SNP	Substitution Type	CCDR	MCR
A→G	Transition	8703	11,102
T→C	Transition	8896	11,228
G→A	Transition	8735	11,420
C→T	Transition	8878	11,622
G→C	Transversion	2817	3523
C→G	Transversion	2730	3559
T→G	Transversion	3274	4108
G→T	Transversion	3367	4158
A→C	Transversion	3316	3964
C→A	Transversion	3339	4175
T→A	Transversion	3734	4428
A→T	Transversion	3685	4397
Total SNPs		61,474	77,684
Total Indels	Insertions and deletions	3647	4609
Total variants		65,121	82,293

## Data Availability

The raw RNA-Seq data is accessible from EMBL-EBI ArrayExpress (accession number E-MTAB-6402). The sequences from the PCR amplified fragments from various rice lines are accessible from the European Nucleotide Archive (study accession PRJEB28811) under the assigned accession numbers ERS2758541-51.

## Supplementary Materials

The following supporting information can be downloaded at: https://www.mdpi.com/article/10.3390/genes14091673/s1. Figure S1: Differential gene expression of the rice Chitin Elicitor Binding (OsCEBiP) and Chitin Elicitor Receptor Kinase (OsCERK) gene family members, analyzed from our transcriptome data; Table S1: Summary of SNP consequence type found in the sheath blight susceptible CCDR and the resistant MCR rice lines; Table S2: The genotype and phenotype results of the top 10 resistant and top ten susceptible individuals of the SB2 double haploid population and the two parents; Table S3: Results of the ANOVA analysis. Includes Marker rank; Table S4: Known *OsWAK91* SNPs extracted from the Gramene Database; Table S5: Name of genes and the list of PCR primers used for genotyping the SNP markers; Table S6: *OsWAK91* SNP Primer Sets and the PCR conditions.
