# Supplementary material for: Loss of a Premature Stop Codon in the Rice Wall-Associated Kinase 91 (WAK91) Gene Is a Candidate for Improving Leaf Sheath Blight Disease Resistance
Source: Genes (Basel). 2023 Aug 24;14(9):1673. doi: 10.3390/genes14091673 (PMC10530950; doi:10.3390/genes14091673)
Supplement: Supplementary file 1 [file genes-14-01673-s001.zip › Supplementary figure-1.pdf]

# Supplementary figure-1

Chitin Elicitor Binding (OsCEBiP) Gene Family

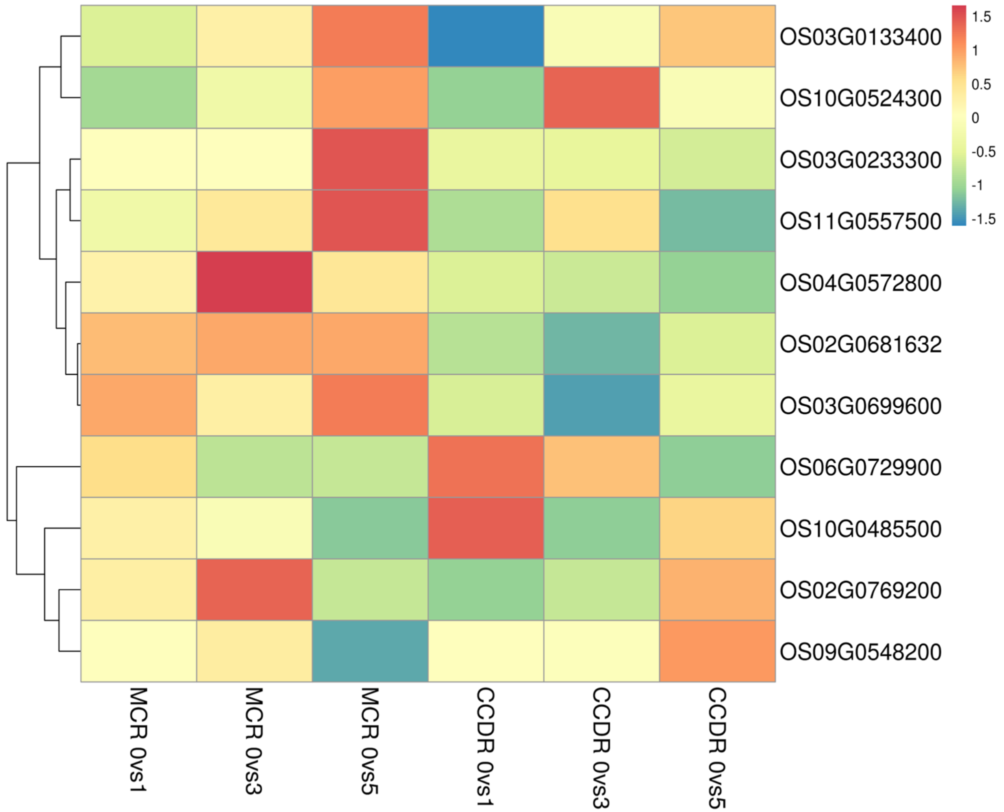


Chitin Elicitor Receptor Kinase (CERK) Gene Family

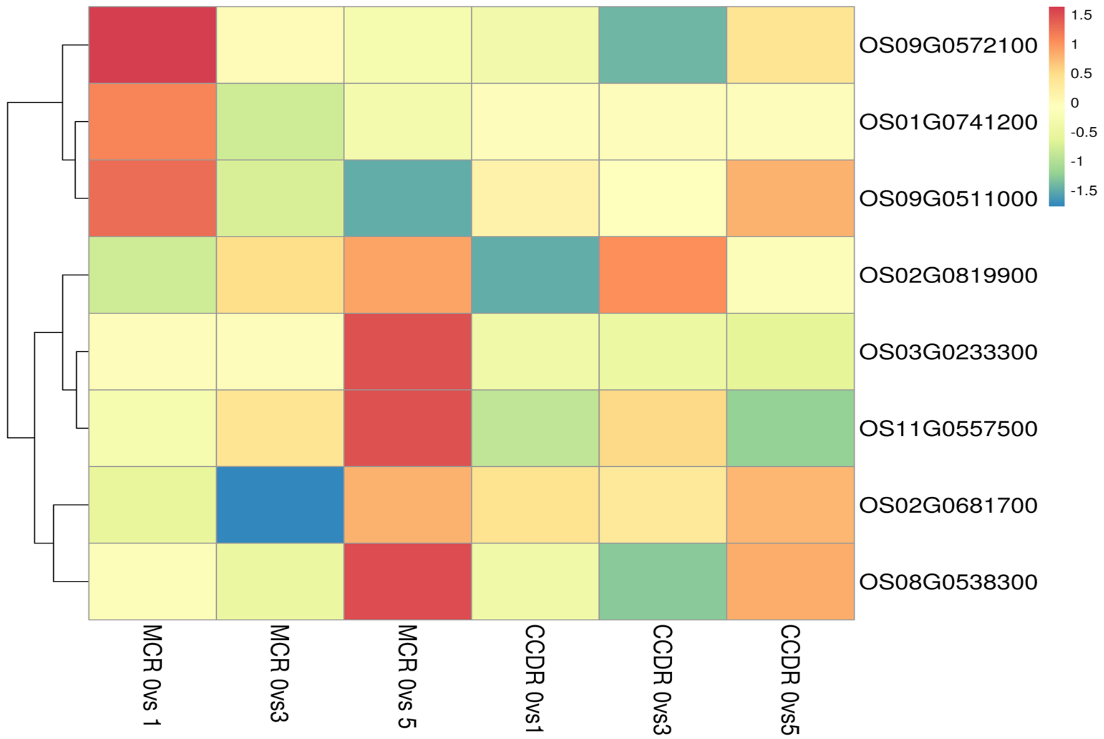


Differential gene expression of the rice Chitin Elicitor Binding (OsCEBiP) and Chitin Elicitor Receptor Kinase (OsCERK) gene family members in the resistant MCR and susceptible CCDR line at Day-1, Day-3 and Day-5 time points after inoculation compared to the Day-0 untreated samples.
