# Supplementary material for: Loss of a Premature Stop Codon in the Rice Wall-Associated Kinase 91 (WAK91) Gene Is a Candidate for Improving Leaf Sheath Blight Disease Resistance
Source: Genes (Basel). 2023 Aug 24;14(9):1673. doi: 10.3390/genes14091673 (PMC10530950; doi:10.3390/genes14091673)
Supplement: Supplementary file 1 [file genes-14-01673-s001.zip › Supplementary Table-1.docx]

**Supplementary Table-1**

Summary of SNP consequence type found in the sheath blight susceptible CCDR and the resistant MCR rice lines. The variant Effect Predictor tool was used to find SNP consequences on gene function and structure based on its overlapping position in the genes identified in the reference *O. sativa japonica* cv Nipponbare reference genome (IRGSP v1.0).

| **SNP Consequence** | **CCDR** | **MCR** |
| --- | --- | --- |
| splice donor variant | 3,207 | 3,457 |
| splice acceptor variant | 1,952 | 2,167 |
| stop gained | 256 | 325 |
| stop lost | 96 | 124 |
| start lost | 26 | 47 |
| missense variant | 12,951 | 18,211 |
| splice region variant | 8,527 | 9,494 |
| synonymous variant | 12,849 | 17,534 |
| stop retained variant | 29 | 33 |
| coding sequence variant | 10 | 8 |
| 5 prime UTR variant | 4,222 | 6,031 |
| 3 prime UTR variant | 17,605 | 20,730 |
| non coding transcript exon variant | 2,389 | 2,694 |
| intron variant | 19,605 | 23,266 |
| non coding transcript variant | 2,854 | 3,311 |
| upstream gene variant | 99,220 | 124,576 |
| downstream gene variant | 110,185 | 138,011 |
| intergenic variant | 887 | 1,103 |
