# Supplementary material for: Loss of a Premature Stop Codon in the Rice Wall-Associated Kinase 91 (WAK91) Gene Is a Candidate for Improving Leaf Sheath Blight Disease Resistance
Source: Genes (Basel). 2023 Aug 24;14(9):1673. doi: 10.3390/genes14091673 (PMC10530950; doi:10.3390/genes14091673)
Supplement: Supplementary file 1 [file genes-14-01673-s001.zip › Supplementary Table-3.docx]

**Supplementary Table-3**

Results of the ANOVA analysis. Includes Marker rank, MSU (v7) and IRGSP (v1.0) rice gene IDs, F value, Bonferroni Hochberg False Discovery Rate (FDR), p values and R-squared values of 130 nsSNP makers genotyped in the 10 most resistant and 10 most susceptible lines of the RiceCAP project’s SB2 double haploid mapping population. N/A in Column 3 suggests missing gene in the IRGSP annotation

| Rank | MSU gene ID | IRGSP gene ID | F value | Raw p value | p value | FDR | R^2^ value |
| --- | --- | --- | --- | --- | --- | --- | --- |
| 1 | LOC_Os09g32860 | N/A | 149.154 | 0 | 0 | 0 | 0.89232 |
| 2 | LOC_Os09g33710 | Os09g0511900 | 61.04 | 0 | 0.00004 | 0 | 0.77227 |
| 3 | LOC_Os09g34180 | Os09g0517600 | 61.04 | 0 | 0.00004 | 0 | 0.77227 |
| 4 | LOC_Os09g36900 | Os09g0540600 | 61.04 | 0 | 0.00004 | 0 | 0.77227 |
| 5 | LOC_Os09g37590 | N/A | 61.04 | 0 | 0.00004 | 0 | 0.77227 |
| 6 | LOC_Os09g37800 | Os09g0550600 | 61.04 | 0 | 0.00004 | 0 | 0.77227 |
| 7 | LOC_Os09g37880 | Os09g0551400 | 61.04 | 0 | 0.00004 | 0 | 0.77227 |
| 8 | LOC_Os09g38700 | Os09g0559900 | 61.04 | 0 | 0.00004 | 0 | 0.77227 |
| 9 | LOC_Os09g38710 | Os09g0560000 | 61.04 | 0 | 0.00004 | 0 | 0.77227 |
| 10 | LOC_Os09g38850 | Os09g0561600 | 61.04 | 0 | 0.00004 | 0 | 0.77227 |
| 11 | LOC_Os09g38970 | Os09g0563250 | 61.04 | 0 | 0.00004 | 0 | 0.77227 |
| 12 | LOC_Os12g10330 | Os12g0204600 | 41.633 | 0 | 0.00052 | 0.00004 | 0.69815 |
| 13 | LOC_Os12g10410 | Os12g0205500 | 41.633 | 0 | 0.00052 | 0.00004 | 0.69815 |
| 14 | LOC_Os12g13100 | Os12g0233100 | 41.633 | 0 | 0.00052 | 0.00004 | 0.69815 |
| 15 | LOC_Os12g15460 | Os12g0256900 | 41.633 | 0 | 0.00052 | 0.00004 | 0.69815 |
| 16 | LOC_Os09g39620 | Os09g0569800 | 37.145 | 0.00001 | 0.00106 | 0.00008 | 0.67359 |
| 17 | LOC_Os12g09710 | Os12g0198900 | 33.113 | 0.00002 | 0.00211 | 0.00016 | 0.56581 |
| 18 | LOC_Os12g10180 | N/A | 33.113 | 0.00002 | 0.00211 | 0.00016 | 0.56581 |
| 19 | LOC_Os09g32020 | Os09g0493500 | 23.457 | 0.00013 | 0.01449 | 0.00097 | 0.48368 |
| 20 | LOC_Os12g06980 | Os12g0167700 | 23.457 | 0.00013 | 0.01449 | 0.00097 | 0.48359 |
| 21 | LOC_Os12g09000 | Os12g0192500 | 16.856 | 0.00066 | 0.07303 | 0.00468 | 0.44991 |
| 22 | LOC_Os12g07950 | Os12g0179800 | 14.722 | 0.00121 | 0.13166 | 0.00807 | 0.352 |
| 23 | LOC_Os06g13040 | Os06g0237400 | 9.778 | 0.00583 | 0.62916 | 0.03699 | 0.3322 |
| 24 | LOC_Os06g15170 | Os06g0262800 | 8.954 | 0.00781 | 0.83613 | 0.04726 | 0.28421 |
| 25 | LOC_Os12g03554 | Os12g0129550 | 7.147 | 0.0155 | 0.9835 | 0.08949 | 0.26383 |
| 26 | LOC_Os12g04660 | Os12g0140700 | 7.147 | 0.0155 | 0.9835 | 0.08949 | 0.26383 |
| 27 | LOC_Os02g34490 | Os02g0549900 | 6.283 | 0.02201 | 0.9835 | 0.10351 | 0.25873 |
| 28 | LOC_Os02g34850 | Os02g0554000 | 6.283 | 0.02201 | 0.9835 | 0.10351 | 0.25873 |
| 29 | LOC_Os02g35210 | Os02g0558400 | 6.283 | 0.02201 | 0.9835 | 0.10351 | 0.25873 |
| 30 | LOC_Os12g07800 | Os12g0177800 | 6.451 | 0.02053 | 0.9835 | 0.10351 | 0.25598 |
| 31 | LOC_Os12g06740 | Os12g0164300 | 6.451 | 0.02053 | 0.9835 | 0.10351 | 0.2426 |
| 32 | LOC_Os08g19694 | Os08g0293300 | 6.193 | 0.02284 | 0.9835 | 0.10361 | 0.2412 |
| 33 | LOC_Os08g20020 | Os08g0296900 | 5.721 | 0.02788 | 0.9835 | 0.11423 | 0.2412 |
| 34 | LOC_Os08g30850 | Os08g0399300 | 5.721 | 0.02788 | 0.9835 | 0.11423 | 0.18705 |
| 35 | LOC_Os08g30910 | Os08g0400200 | 5.765 | 0.02736 | 0.9835 | 0.11423 | 0.18705 |
| 36 | LOC_Os06g22020 | Os06g0325900 | 3.852 | 0.06535 | 0.9835 | 0.20748 | 0.17626 |
| 37 | LOC_Os06g22460 | Os06g0330100 | 3.852 | 0.06535 | 0.9835 | 0.20748 | 0.17626 |
| 38 | LOC_Os06g23530 | Os06g0343100 | 3.852 | 0.06535 | 0.9835 | 0.20748 | 0.17626 |
| 39 | LOC_Os06g28124 | Os06g0475400 | 3.852 | 0.06535 | 0.9835 | 0.20748 | 0.17626 |
| 40 | LOC_Os06g28670 | Os06g0481400 | 3.852 | 0.06535 | 0.9835 | 0.20748 | 0.17626 |
| 41 | LOC_Os06g29700 | Os06g0492800 | 3.852 | 0.06535 | 0.9835 | 0.20748 | 0.17626 |
| 42 | LOC_Os06g29844 | Os06g0494400 | 4.142 | 0.05684 | 0.9835 | 0.20748 | 0.17626 |
| 43 | LOC_Os03g43684 | N/A | 4.142 | 0.05684 | 0.9835 | 0.20748 | 0.1657 |
| 44 | LOC_Os06g31070 | Os06g0507200 | 3.575 | 0.07486 | 0.9835 | 0.23189 | 0.12005 |
| 45 | LOC_Os06g32350 | N/A | 2.204 | 0.15499 | 0.9835 | 0.33529 | 0.12005 |
| 46 | LOC_Os04g10460 | Os04g0183500 | 2.204 | 0.15499 | 0.9835 | 0.33529 | 0.11751 |
| 47 | LOC_Os04g11640 | Os04g0193200 | 2.204 | 0.15499 | 0.9835 | 0.33529 | 0.11751 |
| 48 | LOC_Os04g11970 | Os04g0196200 | 2.204 | 0.15499 | 0.9835 | 0.33529 | 0.11751 |
| 49 | LOC_Os04g15650 | Os04g0227000 | 2.397 | 0.13899 | 0.9835 | 0.33529 | 0.11751 |
| 50 | LOC_Os04g20680 | Os04g0275100 | 2.397 | 0.13899 | 0.9835 | 0.33529 | 0.11751 |
| 51 | LOC_Os04g21890 | N/A | 2.397 | 0.13899 | 0.9835 | 0.33529 | 0.11751 |
| 52 | LOC_Os04g23620 | N/A | 2.397 | 0.13899 | 0.9835 | 0.33529 | 0.11751 |
| 53 | LOC_Os04g23890 | Os04g0304200 | 2.397 | 0.13899 | 0.9835 | 0.33529 | 0.11751 |
| 54 | LOC_Os09g16540 | Os09g0334800 | 2.397 | 0.13899 | 0.9835 | 0.33529 | 0.10953 |
| 55 | LOC_Os09g17600 | Os09g0344800 | 2.397 | 0.13899 | 0.9835 | 0.33529 | 0.10953 |
| 56 | LOC_Os09g17630 | Os09g0345300 | 2.397 | 0.13899 | 0.9835 | 0.33529 | 0.10953 |
| 57 | LOC_Os03g30130 | Os03g0415200 | 2.456 | 0.13451 | 0.9835 | 0.33529 | 0.10907 |
| 58 | LOC_Os03g37720 | Os03g0573500 | 2.456 | 0.13451 | 0.9835 | 0.33529 | 0.10907 |
| 59 | LOC_Os03g39150 | Os03g0588400 | 2.166 | 0.1584 | 0.9835 | 0.33529 | 0.10907 |
| 60 | LOC_Os03g40250 | Os03g0599400 | 2.166 | 0.1584 | 0.9835 | 0.33529 | 0.10907 |
| 61 | LOC_Os08g10560 | Os08g0206500 | 2.214 | 0.15407 | 0.9835 | 0.33529 | 0.10739 |
| 62 | LOC_Os08g12800 | Os08g0224500 | 2.214 | 0.15407 | 0.9835 | 0.33529 | 0.10739 |
| 63 | LOC_Os08g13870 | Os08g0236400 | 2.214 | 0.15407 | 0.9835 | 0.33529 | 0.10739 |
| 64 | LOC_Os03g63110 | Os03g0848400 | 1.475 | 0.24022 | 0.9835 | 0.46294 | 0.07666 |
| 65 | LOC_Os02g42412 | Os02g0636400 | 1.473 | 0.24058 | 0.9835 | 0.46294 | 0.07575 |
| 66 | LOC_Os02g44730 | Os02g0667500 | 1.473 | 0.24058 | 0.9835 | 0.46294 | 0.07564 |
| 67 | LOC_Os02g45160 | Os02g0673100 | 1.473 | 0.24058 | 0.9835 | 0.46294 | 0.07564 |
| 68 | LOC_Os02g45980 | Os02g0684900 | 1.473 | 0.24058 | 0.9835 | 0.46294 | 0.07564 |
| 69 | LOC_Os02g48210 | Os02g0712700 | 1.494 | 0.2373 | 0.9835 | 0.46294 | 0.07564 |
| 70 | LOC_Os08g42930 | Os08g0542200 | 1.203 | 0.2872 | 0.9835 | 0.54439 | 0.06264 |
| 71 | LOC_Os02g58540 | Os02g0832150 | 0.929 | 0.34792 | 0.9835 | 0.63123 | 0.05477 |
| 72 | LOC_Os01g13300 | Os01g0234100 | 0.929 | 0.34792 | 0.9835 | 0.63123 | 0.04907 |
| 73 | LOC_Os04g56250 | Os04g0657600 | 0.898 | 0.35586 | 0.9835 | 0.63654 | 0.04907 |
| 74 | LOC_Os08g35310 | Os08g0454500 | 0.851 | 0.36855 | 0.9835 | 0.64118 | 0.04752 |
| 75 | LOC_Os02g51900 | Os02g0755500 | 0.753 | 0.39685 | 0.9835 | 0.68108 | 0.04513 |
| 76 | LOC_Os02g52060 | Os02g0757400 | 0.615 | 0.44303 | 0.9835 | 0.70947 | 0.04513 |
| 77 | LOC_Os02g02650 | Os02g0118700 | 0.536 | 0.47334 | 0.9835 | 0.70947 | 0.04017 |
| 78 | LOC_Os02g43460 | Os02g0650800 | 0.536 | 0.47334 | 0.9835 | 0.70947 | 0.03305 |
| 79 | LOC_Os02g53970 | Os02g0780200 | 0.536 | 0.47334 | 0.9835 | 0.70947 | 0.02894 |
| 80 | LOC_Os02g54330 | N/A | 0.536 | 0.47334 | 0.9835 | 0.70947 | 0.02894 |
| 81 | LOC_Os02g54500 | Os02g0786000 | 0.533 | 0.47484 | 0.9835 | 0.70947 | 0.02894 |
| 82 | LOC_Os02g55180 | Os02g0795000 | 0.533 | 0.47484 | 0.9835 | 0.70947 | 0.02894 |
| 83 | LOC_Os04g57670 | Os04g0672700 | 0.533 | 0.47484 | 0.9835 | 0.70947 | 0.02875 |
| 84 | LOC_Os04g58720 | Os04g0683800 | 0.533 | 0.47484 | 0.9835 | 0.70947 | 0.02875 |
| 85 | LOC_Os04g58820 | Os04g0685000 | 0.533 | 0.47484 | 0.9835 | 0.70947 | 0.02875 |
| 86 | LOC_Os04g58910 | Os04g0685900 | 0.533 | 0.47484 | 0.9835 | 0.70947 | 0.02875 |
| 87 | LOC_Os04g59060 | Os04g0687300 | 0.508 | 0.48531 | 0.9835 | 0.71668 | 0.02875 |
| 88 | LOC_Os04g59540 | Os04g0691900 | 0.433 | 0.51884 | 0.9835 | 0.74688 | 0.02875 |
| 89 | LOC_Os09g26300 | Os09g0432900 | 0.424 | 0.5234 | 0.9835 | 0.74688 | 0.02743 |
| 90 | LOC_Os04g05030 | Os04g0136900 | 0.343 | 0.56525 | 0.9835 | 0.74778 | 0.02349 |
| 91 | LOC_Os05g37040 | Os05g0442100 | 0.343 | 0.56525 | 0.9835 | 0.74778 | 0.02299 |
| 92 | LOC_Os05g39760 | N/A | 0.356 | 0.55839 | 0.9835 | 0.74778 | 0.02299 |
| 93 | LOC_Os08g36320 | Os08g0465800 | 0.362 | 0.55499 | 0.9835 | 0.74778 | 0.02063 |
| 94 | LOC_Os08g36760 | Os08g0471800 | 0.379 | 0.54578 | 0.9835 | 0.74778 | 0.02063 |
| 95 | LOC_Os09g27570 | Os09g0448100 | 0.379 | 0.54578 | 0.9835 | 0.74778 | 0.02063 |
| 96 | LOC_Os06g44820 | Os06g0658600 | 0.379 | 0.54578 | 0.9835 | 0.74778 | 0.01971 |
| 97 | LOC_Os02g49986 | Os02g0732600 | 0.137 | 0.71588 | 0.9835 | 0.85611 | 0.01937 |
| 98 | LOC_Os02g09820 | Os02g0191500 | 0.137 | 0.71588 | 0.9835 | 0.85611 | 0.01871 |
| 99 | LOC_Os02g10120 | Os02g0194700 | 0.137 | 0.71588 | 0.9835 | 0.85611 | 0.01871 |
| 100 | LOC_Os02g56380 | Os02g0807900 | 0.127 | 0.7261 | 0.9835 | 0.85611 | 0.00789 |
| 101 | LOC_Os02g56480 | Os02g0809100 | 0.143 | 0.70964 | 0.9835 | 0.85611 | 0.00789 |
| 102 | LOC_Os02g57960 | Os02g0826000 | 0.143 | 0.70964 | 0.9835 | 0.85611 | 0.00789 |
| 103 | LOC_Os06g35850 | Os06g0551800 | 0.143 | 0.70964 | 0.9835 | 0.85611 | 0.00789 |
| 104 | LOC_Os06g37500 | Os06g0572300 | 0.143 | 0.70964 | 0.9835 | 0.85611 | 0.00789 |
| 105 | LOC_Os01g52330 | Os01g0721300 | 0.112 | 0.74152 | 0.9835 | 0.85611 | 0.00754 |
| 106 | LOC_Os01g52880 | Os01g0729400 | 0.112 | 0.74152 | 0.9835 | 0.85611 | 0.00754 |
| 107 | LOC_Os01g53420 | Os01g0735900 | 0.112 | 0.74152 | 0.9835 | 0.85611 | 0.00754 |
| 108 | LOC_Os09g25620 | Os09g0424300 | 0.143 | 0.70964 | 0.9835 | 0.85611 | 0.00731 |
| 109 | LOC_Os09g25890 | Os09g0427800 | 0.143 | 0.70964 | 0.9835 | 0.85611 | 0.00731 |
| 110 | LOC_Os02g10900 | Os02g0203500 | 0.139 | 0.71698 | 0.9835 | 0.85611 | 0.00699 |
| 111 | LOC_Os05g40790 | Os05g0486300 | 0.132 | 0.7201 | 0.9835 | 0.85611 | 0.0062 |
| 112 | LOC_Os05g41130 | Os05g0490300 | 0.132 | 0.7201 | 0.9835 | 0.85611 | 0.0062 |
| 113 | LOC_Os05g41290 | Os05g0492200 | 0.132 | 0.7201 | 0.9835 | 0.85611 | 0.0062 |
| 114 | LOC_Os11g19700 | Os11g0302500 | 0.042 | 0.83928 | 0.9835 | 0.89571 | 0.00263 |
| 115 | LOC_Os11g24060 | Os11g0427700 | 0.042 | 0.83928 | 0.9835 | 0.89571 | 0.00263 |
| 116 | LOC_Os11g24180 | Os11g0430000 | 0.042 | 0.83928 | 0.9835 | 0.89571 | 0.00263 |
| 117 | LOC_Os11g24770 | N/A | 0.042 | 0.83928 | 0.9835 | 0.89571 | 0.00263 |
| 118 | LOC_Os11g28950 | Os11g0479100 | 0.047 | 0.83001 | 0.9835 | 0.89571 | 0.00263 |
| 119 | LOC_Os03g53220 | Os03g0743800 | 0.047 | 0.83001 | 0.9835 | 0.89571 | 0.00235 |
| 120 | LOC_Os03g56400 | Os03g0775400 | 0.047 | 0.83001 | 0.9835 | 0.89571 | 0.00235 |
| 121 | LOC_Os03g57160 | Os03g0785300 | 0.047 | 0.83001 | 0.9835 | 0.89571 | 0.00235 |
| 122 | LOC_Os03g58390 | Os03g0798200 | 0.047 | 0.83001 | 0.9835 | 0.89571 | 0.00235 |
| 123 | LOC_Os04g55760 | Os04g0651500 | 0.031 | 0.86218 | 0.9835 | 0.91248 | 0.00172 |
| 124 | LOC_Os02g11820 | Os02g0208900 | 0 | 0.9835 | 0.9835 | 0.9835 | 0.00007 |
| 125 | LOC_Os01g54350 | Os01g0747400 | 0 | 0.9835 | 0.9835 | 0.9835 | 0.00002 |
| 126 | LOC_Os01g54515 | Os01g0748950 | 0 | 0.9835 | 0.9835 | 0.9835 | 0.00002 |
| 127 | LOC_Os01g55050 | Os01g0754500 | 0 | 0.9835 | 0.9835 | 0.9835 | 0.00002 |
| 128 | LOC_Os01g56040 | Os01g0765900 | 0 | 0.9835 | 0.9835 | 0.9835 | 0.00002 |
| 129 | LOC_Os01g57230 | Os01g0780600 | 0 | 0.9835 | 0.9835 | 0.9835 | 0.00002 |
| 130 | LOC_Os01g57900 | Os01g0788900 | 0.001 | 0.97305 | 0.9835 | 0.9835 | 0.00002 |
