# Supplementary material for: Loss of a Premature Stop Codon in the Rice Wall-Associated Kinase 91 (WAK91) Gene Is a Candidate for Improving Leaf Sheath Blight Disease Resistance
Source: Genes (Basel). 2023 Aug 24;14(9):1673. doi: 10.3390/genes14091673 (PMC10530950; doi:10.3390/genes14091673)
Supplement: Supplementary file 1 [file genes-14-01673-s001.zip › Supplementary Table-6.docx]

**Supplementary Table 6**

OsWAK91 SNP Primer Sets and PCR conditions. Primer name with FS and RS suffix were used for amplifying the SNP region and Sanger sequencing. The primer names with suffix FG were used for direct SNP-specific PCR-based genotyping.

| **Primer** | **Sequence** | **Length (nt)** | **Temperature Degree C** | **%GC** |
| --- | --- | --- | --- | --- |
| For PCR and Sanger sequencing | | | | |
| Forward-FS | CAGGTCAAGGAAGAGGGAGG | 20 | 59 | 60 |
| Reverse-RS | AGGAGACATGATTCTCCCTGG | 20 | 58 | 50 |
| For PCR amplification-based genotyping | | | | |
| Forward-FG1 | GAACACTTTCGAGTGTCATCTCCACCAA | 28 | 62 | 46 |
| Forward-FG2 | ACACTTTCGAGTGTCATCTCCACC CG | 26 | 64 | 54 |
